# Longitudinal patterns of adolescent well-being and associations with health-related outcomes in young adulthood: a cohort study using latent growth mixture modelling

**DOI:** 10.1186/s13034-026-01100-w

**Published:** 2026-05-23

**Authors:** Max Herke, Tobias Rähse, Anja Knöchelmann

**Affiliations:** Institute of Medical Sociology, Interdisciplinary Centre for Health Sciences, Medical Faculty of the Martin-Luther-University Halle-Wittenberg, Magdeburger Str. 8, 06112 Halle (Saale), Saxony-Anhalt Germany

**Keywords:** Subjective well-being, Mental health, Adolescence, Longitudinal trajectories, National Educational Panel Study, Germany, Latent class trajectory modelling, Latent growth mixture modelling, Latent class growth analysis

## Abstract

**Background:**

Adolescence represents a sensitive developmental period that can have lasting consequences for later health. This study investigates long-term patterns of subjective well-being during adolescence and their associations with health-related outcomes in young adulthood.

**Methods:**

Drawing on data from the German National Educational Panel Study (NEPS), we use latent growth mixture modelling (LGMM) to identify distinct trajectories of well-being in a cohort of 8,137 adolescents aged 11 to 21 and relate them to childhood social determinants as antecedents and health-related outcomes in young adulthood.

**Results:**

Four distinct classes emerged: a large group with stable high well-being (78.3%), two classes displaying a temporary decrease in subjective well-being earlier around age 15 (11.1%) or later around age 17 (7.8%), and a small catching up group with initially low but improving well-being (2.8%), with the caveat that panel attrition may have limited the identification of an additional persistently adverse trajectory class. Membership in these trajectories was associated with socio-demographic and socio-economic factors. Adolescents from higher-income households and nuclear families were more likely to follow the stable high trajectory, whereas those from single-parent or step-families or attending non-academic schools were more likely to experience early or late drops or belong to the catching up group. Well-being trajectories significantly predicted health-related outcomes in young adulthood (age 22–23), such as self-rated health, body mass index, smoking, and alcohol consumption. The late drop group reported the poorest outcomes, while those in the stable high group reported better overall health and fewer risk behaviours. The catching up group, although initially disadvantaged, approached the levels of their peers, suggesting resilience processes at work.

**Conclusions:**

These findings underscore the importance of adolescence as a sensitive period with lasting implications for health-related outcomes. Supporting adolescent well-being – particularly during critical transition points – may help reduce health inequalities and promote healthier trajectories into adulthood.

**Supplementary Information:**

The online version contains supplementary material available at 10.1186/s13034-026-01100-w.

## Background

Well-being has emerged as an increasingly important construct [1] encompassing multiple dimensions such as mental and physical health, education, living standards, social relationships, and environmental factors [2]. While well-being is vital for health research at any age, it takes on added significance in early and late life. In older age, where illness and functional limitations are common, subjective well-being may better reflect overall health than objective metrics [3]. During adolescence, typically marked by good physical health, well-being is closely related to quality of life and future health outcomes [4, 5] serving as an early indicator of latent issues [6]. As such, longitudinal research is essential to understand its developmental implications [7].

Beyond developmental factors, well-being changes considerably throughout adolescence, with diverging patterns depending on gender and socioeconomic conditions [8–10].

Socioeconomic status (SES) – commonly indicated by parental education, household income, or neighbourhood characteristics – affects well-being both directly and indirectly. The SES influences access to resources, exposure to stressors, and social development opportunities [11, 12]. Adolescents from low-income households may face financial stress, while more privileged adolescents benefit from health-promoting environments and better resources [13]. Social inequality is thus embedded in adolescents’ daily experiences, influencing well-being in significant ways.

Life course epidemiology has shown strong links between childhood and adult health. The “long arm” of childhood and adolescent health emphasises how crucial the early years of life are for long-term physical and mental health. Two major theoretical models explain these associations: the sensitive or critical period model, which highlights how formative experiences during early life affect later health, and the risk accumulation model, which accentuates the cumulative effect of risk factors over time [14]. The former emphasises that exposure to risk factors during a critical period of development usually has irreversible negative consequences for later life, negative influences during a sensitive period are also detrimental in the long term but are more likely to be modifiable and possibly even reversible. Thus, adolescence is considered a sensitive period of life in which physical, emotional, cognitive, and social changes represent demanding developmental challenges on the one hand [5, 6, 15]. On the other hand, successfully navigating these developmental tasks [16, 17] can also be favourable for current and future health, and conversely, good health contributes to meeting these tasks [4, 5, 18]. Applying the sensitive period model to subjective well-being, it can be conceptualized as a positive developmental resource rather than a mere absence of risk [4, 6]. Within this framework, adolescence is viewed as a sensitive period during which the development of well-being is not uniform but characterized by significant heterogeneity, stemming from the varying timing and individual navigation of biological (e.g., puberty) and social developmental tasks [15, 17, 19] and resulting in diverse patterns or classes of well-being trajectories [14]. Specifically, classes reflecting consistently high well-being are hypothesized to act as a formative protective factor that may modify or even reverse the long-term detrimental effects of early socio-economic disadvantages [20, 21]. Conversely, and in line with the risk accumulation model, classes characterized by persistent declines in well-being during this formative stage may represent specific windows of vulnerability that solidify into persistent health inequalities in subsequent (young) adulthood.

Yet, not much is known about the specific mechanisms linking different trajectories of well-being in adolescence to young adulthood and to what extent this association differs between various social groups.

Therefore, the aim of this study is to identify typical classes of adolescent well-being trajectories and relate them to childhood social determinants as antecedents and to well-being, health and health behaviour in young adulthood as health-related outcomes. Drawing on data from the German National Educational Panel Study (NEPS), we investigate the following key questions: Which well-being trajectory patterns can be identified and how are they classified? What are the socio-demographic and socio-economic predictors of trajectory membership? Do these trajectories relate to differences in well-being, health and health behaviour in young adulthood?

### Well-being throughout adolescence

Despite being generally marked by good health [22, 23], increased autonomy, identity exploration, and diverse educational or occupational paths [24], research has frequently documented decline in adolescent well-being. This trend is particularly evident during secondary education, as found in a large-scale study of over 30 European and North American countries [8] as well as in national studies from Germany [25, 26], Spain [27], and the UK [6, 28]. Much of adolescence is spent in school, which represents the most important socialization instance besides the family [29]. Academic performance and career decisions place demands on adolescents, which have been found to contribute to stress and reduced well-being in Finnish longitudinal research [30, 31]. Notably, leaving school marks a pivotal transition point, as young people move from a highly structured environment with limited decision-making to more autonomous settings such as university, vocational training, or voluntary service. This shift tends to relate to improved well-being, as observed in both German [32] and Finnish contexts [33], especially among high-achieving students with strong self-esteem. Taken as a whole, these findings suggest that changes in adolescent well-being are closely tied to age-related developmental processes and to key educational transitions such as leaving school. Both dimensions therefore provide a meaningful temporal structure for modelling heterogeneous well-being trajectories across adolescence.

In the German context, these transitions are particularly shaped by the highly stratified secondary education system and the institutionalized nature of educational transitions. German adolescents face pivotal, track-specific transitions at relatively fixed ages, such as the transition to vocational training or upper secondary school around age 15–16 or to tertiary education between the ages of 18 and 20. Furthermore, the German context is characterized by the strong role of the dual vocational education and training system, a close linkage between qualifications and occupational positioning, and relatively late economic independence compared to international standards [34–36]. This suggests that the developmental window of adolescence might be especially prolonged and sensitive to educational successes or failures. Taken together, these institutional features imply that adolescents in Germany encounter multiple, relatively early and binding educational transitions, which may generate distinct periods of heightened vulnerability or recovery and thereby contribute to heterogeneous well-being trajectories across adolescence and into young adulthood.

Significant gender differences in well-being have been observed, with girls generally experiencing a greater decline in well-being than boys, likely due to different developmental processes and challenges faced during adolescence [27, 37, 38]. A UK-based study found that girls reached earlier and higher peaks in depressive symptoms, suggesting varying psychosocial stresses and developmental trajectories during adolescence. Girls in this cohort experienced stronger increases in depressive symptoms, starting around age 11, with the peak rate of increase at 13.5 years. For boys, the peak rate of increase occurred at 16 years. The highest levels of depressive symptoms were reached at 19.6 years for girls and 20.4 for boys [39].

Research consistently indicates a strong connection between family environment and the overall health and mental well-being of adolescents. Adolescents living in nuclear families (with both biological parents) generally report better well-being than those in single-parent or step-families. They also report fewer health complaints and better health-related quality of life, as found in studies from the United States [40] and Germany [41–44]. This association is further supported by a comparative study of 36 Western societies [45] and European multi-country analyses [46], which found that adolescents in single-parent or step-families tend to report lower life satisfaction compared to those living with both biological parents. Similar results have been documented in the United States [47–49], Germany [50, 51] and Switzerland [52].

Regarding health inequalities, adolescents with a more advantageous SES, typically derived from their parental SES, report more favourable trajectories of well-being and health throughout most of adolescence. This associations have been found in international comparisons across over 30 countries [10, 11] as well as in German national surveys; [37, 53, 54].

However, most findings for changes in subjective well-being throughout adolescence are based on cross-sectional studies and map only smaller time periods, which may reflect biological, psychosocial, and educational transitions typical of adolescence. A life course perspective offers a valuable framework for understanding subjective well-being trajectories. It posits that early social and economic conditions shape later outcomes through cumulative advantage or disadvantage and social stratification [19, 20, 55]. From this view, well-being trajectories reflect not just individual adaptation but also structural inequalities unfolding across the life course. Within this framework, trajectories of well-being are viewed as the mechanisms through which early social determinants are translated into health inequalities in subsequent adulthood.

### Links to later life outcomes

Early life well-being and health have been found to significantly relate to well-being, health outcomes and behaviours in adulthood. Previous research has shown a clear focus on health and well-being in childhood and adverse childhood experiences [56, 57], but adolescence has not been ignored either [58], with research showing lasting effects on physical and mental health. Over the past two decades, researchers have established strong links between early-life conditions and later-life health. Trajectories of prolonged low well-being during adolescence, particularly chronic or increasing patterns, are linked to persistently poorer mental health in young adulthood.

These mental health challenges can also influence health behaviours. Adolescents with lower well-being may engage in risky behaviours, including tobacco and alcohol consumption, physical activity, and dietary habits. Once established during adolescence, these adverse health behaviours tend to solidify into (young) adulthood and are often shaped by socioeconomic factors [59].

Studies have shown that higher socioeconomic status and physical activity in youth are also linked to greater physical activity in adulthood [60, 61]. Similarly, obesity in childhood is a significant predictor of adult obesity, emphasizing the long-term impact of adolescent health behaviours on future well-being [62].

### Current study

This study aims to deepen our understanding of the relevance of adolescent well-being for later health-related outcomes by identifying and analysing typical trajectories of well-being across adolescence. The analysis draws on rich longitudinal data from the German National Educational Panel Study (NEPS), which follows a representative cohort of students in Germany [63], although, as is common in long-term longitudinal studies, affected by panel attrition. Students were surveyed annually beginning in fifth grade (around age 10–11), allowing for the identification of complex and nonlinear age-based well-being trajectories across adolescence, while explicitly accounting for the transition of leaving school as a key developmental event. Subjective well-being will be assessed through validated self-reported measures, including domains such as life satisfaction, health satisfaction, and emotional well-being. Using latent class growth analysis, it summarises 54,914 observations of 8,178 students aged 11 to 21 into distinct classes of trajectories. These classes provide insight into how adolescents manage the developmental demands and psychosocial stressors of this sensitive life stage.

Beyond describing these trajectories, the study examines to what extent socio-demographic and socio-economic characteristics predict class membership. It considers early-life structural factors, including household income, parental education, and family structure, to assess how social conditions shape well-being across adolescence. By doing so, it contributes to our understanding of how early disadvantage may lead to divergent developmental pathways, thereby reinforcing or mitigating long-term health inequalities.

Finally, the study explores whether distinct adolescent well-being trajectories are associated with differences in health-related outcomes in young adulthood, including well-being, self-rated health, and health-related behaviours. Subjective well-being thus serves two analytically distinct roles in this study. It is first utilized to identify longitudinal trajectories throughout adolescence (ages 11–21), and subsequently examined as one of several health-related outcomes in young adulthood (ages 22–23). Importantly, the aim is not to test simple continuity in well-being levels, but to assess whether different developmental patterns of well-being, such as stable, declining, or improving trajectories, are associated with varying later health-related outcomes, as these trajectories capture differences in the timing, stability, and direction of change across adolescence, while also accounting for socio-demographic background characteristics.

We will address three central research questions. (1) Which distinct patterns (i.e., latent classes) of trajectories of well-being during adolescence can be found and described? (2) Do socio-demographic and -economic characteristics determine the trajectories of well-being adolescents are likely to experience? (3) What associations do trajectories of well-being during adolescence have on health-related outcomes in young adulthood?

We approach these questions with the following general and non-exhaustive assumptions. There will be identifiable and distinct well-being trajectories among adolescents, e.g., consistently high, consistently low, and fluctuating patterns, which can be statistically classified. Individuals with more positive well-being trajectories during adolescence, e.g., those experiencing longer periods of better well-being, will engage in healthier behaviours and report better health-related outcomes in young adulthood compared to others. Adolescents from disadvantageous socio-economic backgrounds will experience more negative trajectories of well-being compared to their advantaged counterparts, e.g., lower overall well-being scores and greater declines over time. Furthermore, we assume that individuals will tend to experience different developments in well-being depending on gender and family type. And finally, the association between trajectories of well-being on health-related outcomes in young adulthood remains significant even after controlling for socio-economic factors, suggesting an independent effect of these trajectories.

Ultimately, this study aims to underline the importance of adolescence as a whole for later manifestations of health-related disparities, by providing a novel, comprehensive analyses of heterogeneous subjective well-being trajectories across the entire period of adolescence and linking these developmental patterns to health-related outcomes in young adulthood, independent of socio-economic and socio-demographic factors.

## Methods

### Data

We use data from the German National Educational Panel Study (NEPS), which collects longitudinal data on the development of competencies, educational processes, educational decisions and educational returns over the entire lifespan [63]. NEPS comprises six distinct cohorts sampled from different age groups. This study focuses on data from Starting Cohort 3 (SC3, “Paths through Lower Secondary School”), which began with a representative sample of German 5th graders in the 2010/2011 school year and was followed up annually or biennially[64].

NEPS employed a hierarchical two-stage sampling design. In the first stage, schools were selected as primary sampling units using probability proportional to size sampling, based on comprehensive school directories from the 2008/2009 school year provided by the Federal Statistical Office of Germany. This selection was stratified by factors such as school type, federal state, regional classification and funding institution, ensuring a representative mix. The final sample comprised 365 schools, including 240 regular schools, 65 special-needs schools, and 60 schools in the migration supplement.

In the second stage, within selected regular schools, two classes were randomly chosen when more than two were present, and all classes were included otherwise. In special-needs schools, all classes were included. All students from these classes were invited to participate, forming the main sample. Additionally, a migrant supplement targeted students with backgrounds from Turkey and the former Soviet Union. The initial SC3 sample consisted of 6,112 individuals from fifth grade. In Wave 3, a refreshment sample of 2,205 seventh-grade students was added to compensate for panel attrition and to maintain cohort representativeness, following the same sampling design and accounting for differing primary-to-secondary transition timings in Berlin and Brandenburg, where primary schooling lasts six years. Data collection involved paper-based questionnaires completed by students during class time, along with telephone interviews with their parents and paper surveys distributed to teachers and school heads. Follow-ups were conducted annually or biennially through these methods, with telephone interviews utilised for students who transferred schools or left the original cohort. Information from parents, teachers and school heads was updated at intervals of one to three years. Unique identification codes assigned to participants, classes, schools, and each wave facilitated coherent data integration. This research primarily analyses variables derived from student questionnaires, supplemented with data from parent interviews at the student level.

### Variables

For the latent trajectory models, subjective well-being was specified as the repeatedly measured outcome variable. Age was used as the developmental time scale to model change in well-being across adolescence. Leaving school was included as a time-varying indicator capturing a key developmental transition that may coincide with changes in well-being trajectories. To predict membership to the classes of latent trajectories, we draw upon four socio-demographic and -economic indicators, specifically gender, school type, family type, and parental income. Finally, we assess the associations of latent trajectories on five outcomes, subjective well-being, self-rated health, body mass index (BMI), smoking status, and alcohol consumption.

Subjective well-being is measured in every survey wave and is constructed as an index of several items, adapted from the Personal Well-being Index – School Children [65]. These items ask “How satisfied are you…” on a 0 to 10 numeric scale covering several domains, i.e., life satisfaction (“…with your life overall at the present?”), standard of living (“…with what you have? Think of money and things that you own.”), personal health (“…with your health?”), family (“…with your family life?”), personal relationships (“…with your group of friends and acquaintances?”) and school (“…with your situation at school?”). Before leaving school, subjective well-being was assessed using six domains, including school satisfaction. After school leaving, the school-related item was omitted, resulting in a five-item index. Internal consistency was good for both versions of the scale (in-school: Cronbach’s α = 0.81, McDonald’s ω = 0.81; post-school: α = 0.80, ω = 0.80). All scales are transformed to a range of 0 to 100, with higher values indicating better subjective well-being. Since no universal definition of adolescence exists, and while the WHO’s age range of 10 to 19 was used as a general reference, adjustments were made to fit the study’s requirements. Observations below age 11 were excluded due to an insufficient number of cases (*n* = 54), with the transition into secondary education considered a more relevant marker for entering adolescence. A slightly higher upper age limit of 21 was chosen to ensure that most individuals had completed secondary education, as this represents a significant life event and in Germany, students typically leave upper secondary education between the ages of 18 and 20.

Leaving school marks the departure from regular school and the transition to any other context that is not lower or upper secondary school, including vocational training, military service, university, unemployment, etc. This transition is coded with a dummy variable (0 = “attends school”, 1 = “left school”).

Socio-demographic and -economic variables include gender, family type, school type, and parental income. For gender, we use the adolescents’ self-reported data (0 = “male”, 1 = “female”). Family type is based on adolescents’ data on persons living in the private household. We differentiate adolescents living in traditional families with both their biological mother and father (1 = “nuclear families”), and other family types, including single-parent families and step-families (0 = “other family types”). Regarding school type, the German secondary education system features a hierarchical structure with multiple tracks. We dichotomize this into educational tracks that provide higher secondary education including a university entrance qualification (1 = “academic track”) and those that end after lower secondary education, usually after grade 9 or 10 (0 = “non-academic track”). Parental income is assessed using data from the parent interviews on household income and household size to determine the equivalised household income using the OECD square root scale equivalization [66]. Median splits were performed at the baseline measure (1,500€) differentiating high (1 = “above 1,500 € equivalised household income”) and low (0 = “at most 1,500 € equivalised household income”) parental income. Parental education, household income and family structure are not measured in all survey waves. Data for these measures at any given time point were carried over observations as following time points.

Health-related outcomes include – besides subjective well-being itself – self-rated health, BMI, smoking status and alcohol consumption. All these outcomes were measured in the outcome assessment wave, i.e., survey wave 12 in 2021/2022, at a mean age of 22. Self-rated health was assessed using a 5-point scale, which is dichotomised to differentiate good health (0 = “very good”, “good” and “moderate”) and poor health (1 = “poor” and “very poor”). The BMI is based on the adolescents’ self-reported data on weight and height. After omitting implausible values (i.e., values below 10 and above 100), the measures for BMI range from 12.5 to 49.7. Smoking status is based on the question “Do you currently smoke - even if only occasionally?”. The dichotomised variable differentiates current smokers (1 = “yes, daily” and “yes, occasionally”) and non-smokers (0 = “no, not anymore” and “never smoked”). Alcohol consumption was surveyed using a 6-point scale for the question “How often do you drink alcoholic beverages? When answering, please think of the average over the last 12 months.” We dichotomised this to differentiate low alcohol consumption (0 = “never”, “once a month or less” and “two to three times a month”) and problematic alcohol consumption (1 = “once a week”, “multiple times a week” and “daily”), with weekly alcohol consumption being a common indicator for regular alcohol use in international studies on youth and adolescent health behaviours [67].

### Statistical analysis

The statistical analysis follows a comprehensive approach including several steps. Starting with the description of the sample, followed by a latent trajectory analysis from which classes of typical trajectories of well-being throughout adolescence are derived. After providing a description of the classes, we also examine predictors of class membership using multinomial regression models. To examine the associations of different trajectories on later health, we also conduct several linear and logistic regression analyses on multiple health-related outcomes with the classes as predictors. Finally, we conduct a series of sensitivity analyses to assess the robustness of the results.

First, we provide a description of the sample at baseline, which incorporates all students included in the first survey wave in 2010/2011 and students from the additional sample drawn for the third survey wave in 2012/2013. We also provide a description of individuals surveyed in the twelfth survey wave in 2021/2022, which is referred to as the outcome assessment wave. We also provide an assessment of attrition.

Second, we identify typical trajectories of subjective well-being for adolescents aged 11 to 21 using latent trajectory modelling. Latent trajectory modelling is a statistical method used to identify and analyse patterns of change over time within a population, focusing on the trajectories of individuals’ outcomes across multiple time points and identifying distinct subgroups with similar patterns of change. This approach is particularly well suited to the present study, as it allows for the identification of heterogeneous and potentially non-linear patterns across the entire period of adolescence, rather than assuming a single average trajectory. Depending on the study design, different methods are used for latent trajectory modelling, two of which are used and presented here. Latent Class Growth Analysis (LCGA) assumes that there are distinct, non-overlapping subgroups within the population, with each subgroup following the same trajectory. While LCGA is useful for exploring broad trajectory patterns, it does not account for individual differences within classes, as it relies solely on fixed effects. In contrast, Latent Growth Mixture Modelling (LGMM) extends this framework by allowing for within-class variability through random effects, thereby capturing individual-level differences in developmental change. Given the study’s aims, LGMM provides a more flexible and nuanced understanding of the data by considering both class-level as well as individual-level variability [68].

We fitted a series of six latent trajectory models using the R package lcmm [69]. The first three models used LCGA, where we explored the effects of age, age squared, and age cubed, along with the transition of leaving school, on well-being, without accounting for individual variability within classes. The next three models incorporated the same predictors, but added a random intercept to account for individual differences, utilizing LGMM. All models include the dummy variable for attending school as a predictor, to model the known association of the transition of leaving school on well-being [32, 33].

For each of these six models, we tested the fit across 1 to 7 latent subgroups to determine the optimal number of distinct trajectories within the sample. We compared the Bayesian Information Criterion (BIC), the Sample-Adjusted Bayesian Information Criterion (SABIC), and entropy, as well as the plots of observed and predicted values by class for practical significance to determine the best model fit [68]. Likelihood ratio tests were also considered during the model selection process. Consistent with previous findings in mixture modelling, these tests tended to support additional classes as sample size increased. To avoid overfitting and substantively implausible solutions with very small classes, model selection was based on converging evidence from information criteria, entropy, and substantive interpretability. We followed the framework to construct and interpret latent class trajectory modelling proposed by Lennon et al. [70] and reported as many items as possible and sensible from the GRoLTS Checklist (Guidelines for Reporting on Latent Trajectory Studies, [71]).

Third, after determining the optimal fitting latent trajectory model, we examined possible predictors of class membership using multinomial logistic regression. In this cross-sectional analysis we draw upon the baseline data and examine the relevance of gender, school type, family type, and parental income as predictors of class membership, using the most prevalent class as a reference.

Fourth, we examined the predictive value of trajectories of well-being for health-related outcomes in young adulthood using linear and logistic regressions. Specifically, we analysed the associations of class membership and thus of the experiences of subjective well-being throughout adolescence on later subjective well-being, self-rated health, smoking, alcohol consumption, and BMI. To distinguish this influence from the predictors of the trajectories, gender, school type, family type and income were controlled for here.

All data handling and analyses were conducted using R Version 4.3.2 [72].

## Results

### Sample description

A description of the distribution of socio-demographic and -economic characteristics as well as the health-related outcomes at baseline and at the final outcome assessment wave is provided in Table 1. The baseline consists of 6,112 students in 5th grade in the first survey wave in 2010 and the additional sample of 2,205 students in 7th grade in the third survey wave in 2012.

**Table 1:** Descriptive characteristics of the analytical sample at baseline (*n* = 8,317) and at final outcome assessment wave (*n* = 2,924)

	Baseline (initial sample wave 1 and additional sample wave 3, *n* = 8,317)		Outcome assessment (wave 12, *n* = 2,924)	
	*n*	%	*n*	%
Demographics				
Gender				
Female	3,981	47.9	1,461	50.0
Male	4,332	52.1	1,462	50.0
Missing	4	0.0	1	0.0
School Type				
Academic track schools	3,399	40.9	1,684	57.6
Non-academic track schools	4,036	48.5	1,136	38.9
Missing	882	10.6	104	3.6
Family type				
Nuclear family	5,555	66.8	2,162	73.9
Other family types	2,599	31.2	749	25.6
Missing	163	2.0	13	0.4
Parental income^a^				
High (above 1,500 €)	2,608	31.4	1,526	52.2
Low (at most 1,500 €)	2,642	31.8	643	22.0
Missing	3,067	36.9	755	25.8
Health indicators				
Subjective well-being (mean ± sd)		84.2 ± 16.2		82.0 ± 8.9
Missing	1,481	17.8	1	0.0
Self-rated health				
Good	6,166	74.1	2,494	85.3
Poor	1,046	12.6	430	14.7
Missing	1,105	13.3	0	0.0
BMI				
High (over 25)	-	-	673	23.0
Low (at most 25)	-	-	2,204	75.4
Missing	-	-	47	1.6
Smoking status				
Yes	-	-	720	24.6
No	-	-	2,204	75.4
Missing	-	-	0	0.0
Problematic alcohol consumption				
Yes	-	-	754	25.8
No	-	-	2,169	75.4
Missing	-	-	1	0.0

Sample comprises cohort three (“Fifth Grade”) from the National Educational Panel Study (NEPS). Baseline combines wave 1 and additional cases from wave 3. Subjective well-being ranges from 0 (low) to 100 (high). BMI and health behaviour variables were only assessed in the outcome wave. Subjective well-being measured on a 0–100 scale

Sd: standard deviation

^a^Equivalised net household income based on OECD square root scale

The gender distribution is roughly equal, slightly more adolescents attended non-academic tracks rather than academic tracks, roughly one third lived in traditional nuclear families with both parents, and the median equivalised household income was 1,500€. Approximately 10% of the information on the type of school is missing, as this could either not be definitively determined or the adolescents attended special schools that could not be assigned to any of the other tracks. About one third of income data is missing, as many parents either did not participate in the separate parental interviews or did not disclose this information. However, missing data here does not affect the latent trajectory modelling, since it only relies on the measures of subjective well-being throughout the survey waves.

At baseline, adolescents that provided information reported on average high levels of subjective well-being (mean = 84.1; sd = 16.2) and over 85% reported good overall health.

Survey wave 12 from 2022 was drawn upon as the outcome assessment wave in which the respondents were about 23 years old and retained 2,924 individuals from the original sample, leading to a panel mortality of around 64%. Regarding health-related outcomes, at this follow-up individuals also reported high levels of subjective well-being (mean = 82.0; sd = 8.9). The proportion of those reporting good health increased as well, which might point towards a higher panel mortality for those with lower subjective well-being and poor self-rated health at baseline. About one quarter of individuals can be considered overweight with a BMI of over 25, one quarter reports to smoke at least occasionally, and one quarter consumes alcohol at least once a week and thus exhibits problematic patterns of alcohol consumption.

Compared to baseline, the gender distribution stayed equal, but the proportion of individuals that attended or completed an academic track, that live or grew up in traditional nuclear families, or live or grew up in families with an above median income increased substantially. Thus, panel attrition is linked to indicators of unfavourable socio-economic status suggesting that missingness is associated with observed socio-economic characteristics. Accordingly, analyses were conducted under the Missing at Random (MAR) assumption, taking these characteristics into account.

### Latent trajectories of subjective well-being

We examined two different types of latent trajectory models, the first one being a LCGA without additional random effects, and the second one being a LGMM including random intercepts to account for additional variance within each latent class. A full overview of all tested latent trajectory models as described before is provided in the additional files [see Additional Files 1–12].

After comparing all models, we decided on the LGMM with a cubic structure to best represent the observed values while also providing excellent model fits. Table 2 provides an overview of central indicators relevant for the final model selection. Likelihood ratio tests comparing consistently favoured solutions with additional classes. However, these solutions mainly resulted in further fragmentation into several very small classes with irregular and difficult-to-interpret trajectories. We therefore retained the four-class solution as the most parsimonious and substantively meaningful representation. While the maximum likelihood, BIC and SABIC indicated improvements in model fit up to the seven-class solution, entropy pointed towards the models with three or four classes providing the best at distinguishing classes. Since both models allowed for good interpretability of the classes, we opted for the four-class solution with slightly better entropy. However, all models displayed similar patterns and strong overlaps in the observed trajectories.

**Table 2:** Latent growth mixture model. Characteristics and criteria for increasing number of classes (K = 1–7) using cubic structure model including transition dummy and random intercept

G	loglik	npm	BIC	SABIC	Entropy	Class membership (%)						
						1	2	3	4	5	6	7
1	-217455.451	6	434964.958	434945.891	1	100%						
2	-216322.012	12	432752.134	432,714	0.709	83.0%	17.0%					
3	-214761.749	18	429685.663	429628.463	0.779	79.5%	3.6%	16.9%				
4	-214369.05	24	428954.321	428878.054	0.794	78.3%	11.1%	7.8%	2.8%			
5	-214082.029	30	428434.334	428338.999	0.721	3.5%	18.2%	66.6%	9.0%	2.7%		
6	-213908.91	36	428142.151	428027.75	0.708	4.3%	3.8%	6.4%	60.3%	23.3%	1.9%	
7	-213799.878	42	427978.142	427844.674	0.723	60.9%	1.8%	3.7%	7.7%	21.6%	1.2%	3.0%

G: number of groups; loglik: log-likelihood; npm: number of parameters; BIC: Bayesian Information Criterion; SABIC: Sample-Adjusted BayesianInformation Criterion; % G: percentage of identified groups in given model

We identified four distinct classes. The first and with 78.3% of all cases most prevalent class includes individuals with relatively *stable high* subjective well-being throughout adolescence, starting and ending of with overall very high levels of subjective well-being, though exhibiting a slight downwards trajectory over time. The second class with 11.1% of all individuals has similar baseline and endpoints, but with an *early drop* in subjective well-being, with the lowest levels reported around age 15. The third class with 7.8% of all cases follows a similar trajectory, but with a *late drop* and the worst subjective well-being at age 17. Finally, the smallest class with only 2.8% of cases (which is discussed in more detail below), starts off with extremely low levels of subjective well-being, but is *catching up* throughout adolescence and reaches levels comparable to the other groups at age 21. The predicted values for each class match the observed values well, except for the later points in time for class three, where the predicted values remain significantly below the observed values.

**Fig. 1:**
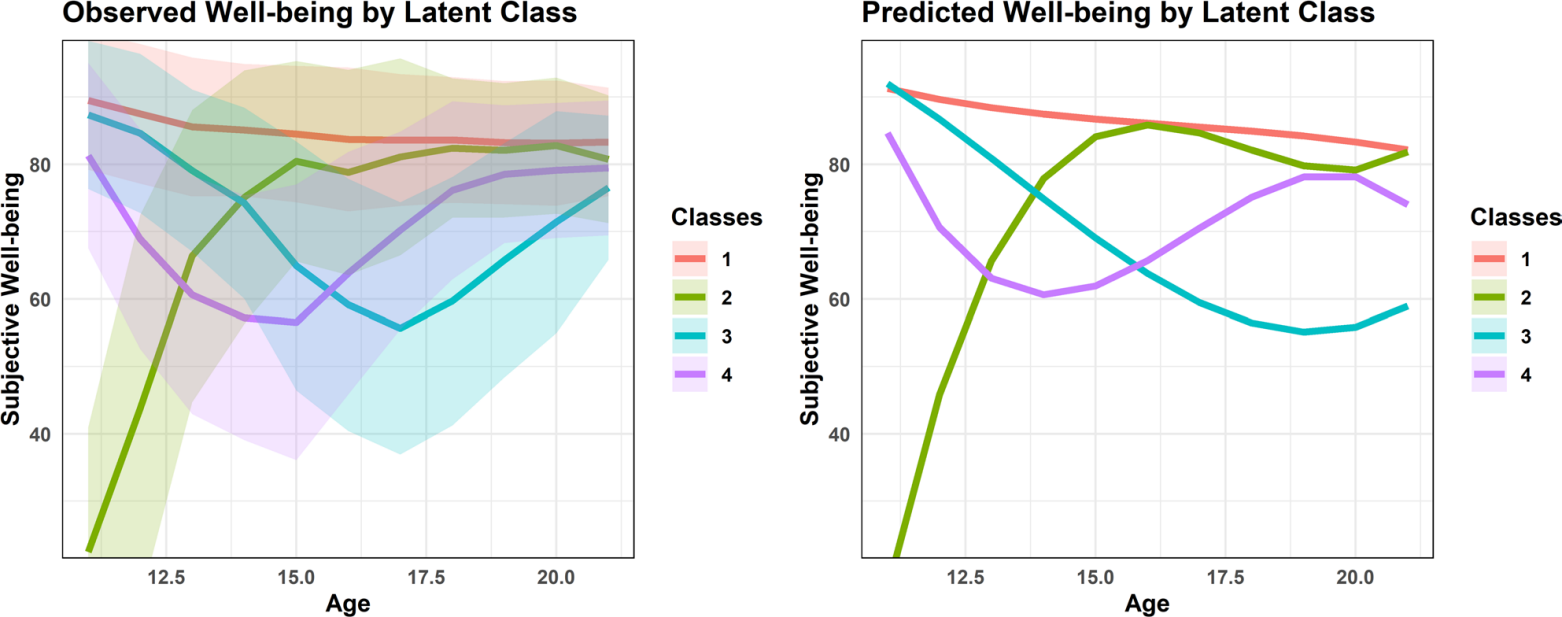
Latent class growth analysis. Observed values vs. predicted values by class membership. Number of subjects: 8,137. Number of observations: 54,914. Model fit statistics given in Table 2

Figure 1 provides an easily interpretable illustration of the trajectories of subjective well-being of these classes. The observed values of subjective well-being for each class at each age are displayed on the left. A complete overview of all model coefficients is provided in Table 3. All coefficients show statistical significance and the derived predicted values for each class are presented on the right in Fig. 1.

**Table 3:** Latent growth mixture model. Overview of model coefficents.

	Coef	SE	*p*-value
Constant			
Class 1 *stable high*	91.2871	0.22264	0.000***
Class 2 *early drop*	84.66374	0.85062	0.000***
Class 3 *late drop*	91.99505	0.84983	0.000***
Class 4 *catching up*	16.85496	1.77437	0.000***
Time			
Class 1 *stable high*	-1.82055	0.16966	0.000***
Class 2 *early drop*	-17.67497	0.64822	0.000***
Class 3 *late drop*	-4.86421	0.65431	0.000***
Class 4 *catching up*	34.05451	1.27968	0.000***
Time²			
Class 1 *stable high*	0.22392	0.03859	0.000***
Class 2 *early drop*	3.89178	0.14127	0.000***
Class 3 *late drop*	-0.46479	0.14738	0.002**
Class 4 *catching up*	-5.34642	0.26447	0.000***
Time³			
Class 1 *stable high*	-0.0133	0.00253	0.000***
Class 2 *early drop*	-0.22309	0.00876	0.000***
Class 3 *late drop*	0.06209	0.0093	0.000***
Class 4 *catching up*	0.2591	0.01588	0.000***
Transition			
Class 1 *stable high*	2.87553	0.23563	0.000***
Class 2 *early drop*	5.44792	0.65832	0.000***
Class 3 *late drop*	21.16667	1.0405	0.000***
Class 4 *catching up*	4.09398	1.27516	0.001**

Number of subjects: 8,137. Number of observations: 54,914. Model includes cubic time terms and a transition dummy. Estimates are unstandardised. See Table 2 for model selection criteria

An examination of the posterior probability of classification given in Table 4 indicates great accuracy in class separation, with slight deductions for classes two and three, as the *early drop* and *late drop* classes both had about ten to twelve percent probability of instead belonging to class one *stable high*.

**Table 4:** Matrix of the average latent class posterior probability from four-class solution

	Prob. Class 1	Prob. Class 2	Prob. Class 3	Prob. Class 4
Class 1 stable high	0.914	0.036	0.036	0.015
Class 2 *early drop*	0.107	0.790	0.071	0.032
Class 3 *late drop*	0.117	0.064	0.816	0.004
Class 4 *catching up*	0.059	0.070	0.005	0.866

Diagonal values indicate the average posterior probability of correctly assigning individuals to their respective class. Off-diagonal values reflect potential misclassification. High diagonal values support the reliability of the four-class solution

### Antecedents of trajectories of well-being

We examined whether several socio-demographic and -economic characteristics have any predictive value of the trajectory of subjective well-being the adolescents are most likely to experience. The first class with *stable high* subjective well-being was chosen as the reference category. Table 5 provides an overview of the extent to which the various predictors influence the probability of experiencing the diverging trajectories of other latent classes.

**Table 5:** Multinomial logistic regression analysis: predictors of well-being paths (reference class = 1 *stable high*)

	Class 2 early drop		Class 3 late drop		Class 4 catching up	
	OR (95%-CI)	*p*-value	OR (95%-CI)	*p*-value	OR (95%-CI)	*p*-value
Constant	0.27 (0.23–0.33)	0.000***	0.19 (0.15–0.24)	0.000***	0.04 (0.03–0.07)	0.000***
Gender ^Ref=female^	1.02 (0.86–1.22)	0.807	0.69 (0.56–0.86)	0.001**	1.48 (1.02–2.15)	0.039*
School ^Ref=low^	0.69 (0.57–0.83)	0.000***	0.8 (0.64–1.01)	0.056	0.47 (0.32–0.71)	0.000***
Family ^Ref=non−nuclear family^	0.66 (0.54–0.79)	0.000***	0.77 (0.61–0.98)	0.031*	0.62 (0.42–0.91)	0.016*
Parental income ^Ref=low^	0.9 (0.75–1.08)	0.260	0.77 (0.61–0.96)	0.021*	1.34 (0.92–1.97)	0.130

Odds ratios (OR) and 95% confidence intervals (CI) are reported. All predictors were measured at baseline. Statistical significance is indicated by * p < 0.05, ** p < 0.01, and *** p < 0.001.

Class two, marked by an *early drop* in subjective well-being is less likely for students attending academic track schools and those living in traditional nuclear families with both parents. Class three, characterised by a *late drop* in subjective well-being, demonstrates similar associations for school tracks and family types, though to a lesser extent. However, experiencing the *late drop* of class three is more likely for females or those living with parents earning a below median income. Finally, the *catching up* in subjective well-being of class four is strongly associated with being male, attending a non-academic track school and living in a single-parent or step-parent family. There is also an uncertain indication that group four may be more likely to be associated with high parental income.

### Consequences of trajectories of well-being

Table 6 provides an overview of how socio-demographic and -economic characteristics as well as health-related outcomes are distributed amongst the different classes for the 2,924 cases remaining at the outcome assessment wave, i.e., the twelfth wave of the NEPS from 2022, at which point all individuals had left adolescence behind and were on average 23 years old. Comparing the proportions of classes in this wave with those from the longitudinal analyses, those belonging to class one with *stable high* subjective well-being are also slightly more likely to remain in the panel. The patterns of how socio-demographic and -economic characteristics have previously predicted class membership (see Table 5) are also reflected in this endpoint.

**Table 6:** Descriptive characteristics of well-being classes at outcome wave (wave 12, *n* = 2,924)

Total	Class 1 stable high	Class 2 early drop	Class 3 late drop	Class 4 catching up
	*n* (%)	*n* (%)	*n* (%)	*n* (%)
	2,341 (80.1)	300 (10.3)	206 (7.0)	77 (2.6)
	**%**	**%**	**%**	**%**
Demographics				
Gender				
Female	49.9	51.7	51.5	42.9
Male	50.1	48.3	48.5	57.1
Missing	0.0	0.0	0.0	0.0
School Type				
Academic track schools	59.2	51.7	53.9	42.9
Non-academic track schools	37.1	45.0	45.6	51.9
Missing	2.7	0.5	0.5	3.3
Family type				
Nuclear family	76.0	65.3	67.5	65.3
Other family types	23.7	33.0	33.5	32.5
Missing	0.3	1.7	0.5	0.0
Parental income^a^				
High (above 1500)	54.1	46.0	42.7	45.5
Low (at most 1500)	20.7	27.7	26.2	26.0
Missing	25.2	23.3	31.1	28.6
Health Outcomes				
Subjective well-being (mean ± sd)	82.9 ± 8.3	80.2 ± 9.1	76.5 ± 10.8	79.5 ± 10.2
Missing	0.0	0.0	0.0	1.3
Self-rated health				
Good or better	87.9	76.7	72.3	74.0
Poor or worse	12.1	23.3	27.7	23.3
Missing	0.0	0.0	0.0	0.0
BMI				
High (over 25)	21.7	30.0	26.2	27.3
Low (at most 25)	77.0	67.3	70.9	70.1
Missing	1.3	2.7	2.9	2.6
Smoking				
Yes	23.2	33.3	28.2	24.7
No	76.8	66.7	71.8	75.3
Missing	0.0	0.0	0.0	0.0
Alcohol				
Yes	26.9	22.0	21.8	18.2
No	73.1	78.0	78.2	81.8
Missing	0.0	0.0	0.0	0.0

Sample comprises wave 12 of cohort three (“Fifth Grade”) from the National Educational Panel Study (NEPS). Subjective well-being measured on a 0–100 scale

Sd: standard deviation

^a^Equivalised net household income based on OECD square root scale

Class one not only is associated with the more beneficial characteristics on average in terms of school type, family form and parental income, but also largely with better health-related outcomes. Those experiencing *stable high* subjective well-being throughout adolescence also are most likely to report higher levels of subjective well-being and better self-rated health later on, to not be overweight and to not smoke. However, they are more likely to report problematic alcohol consumption. More detailed analyses of differences are provided below.

Subjective well-being (see Table 7) is lower in those experiencing the *late drop* in well-being throughout adolescence. Those experiencing an *early drop* or *catching up* report only slightly lower levels of subjective well-being, almost reaching the levels of the *stable high* class. Furthermore, the positive association of living or growing up in nuclear families with both parents and higher parental income is significant, although of almost negligible magnitude.

**Table 7:** Linear regression analysis: trajectories of well-being as predictors of health and health behavior

	Subjective well-being		BMI	
	Coef (95%-CI)	*p*-value	Coef (95%-CI)	*p*-value
Constant	80.42 (79.41–81.43)	0.000***	24.61 (24.16–25.06)	0.000***
Class ^Ref=class 1 *stable high*^				
Class 2 *early drop*	-2.91 (-4.11–1.71)	0.000***	0.51 (-0.03-1.05)	0.066
Class 3 *late drop*	-7.3 (-8.81–5.8)	0.000***	0.96 (0.28–1.64)	0.006**
Class 4 *catching up*	-2.12 (-4.54-0.3)	0.086	-0.73 (-1.82-0.36)	0.189
Gender ^Ref=male^	0.21 (-0.53-0.95)	0.585	-1.25 (-1.58–0.92)	0.000***
School ^Ref=low^	-0.15 (-0.93-0.64)	0.715	-0.88 (-1.23–0.53)	0.000***
Family ^Ref=non−nuclear family^	2.36 (1.49–3.23)	0.000***	-0.33 (-0.72-0.05)	0.091
Parental income ^Ref=low^	0.96 (0.15–1.76)	0.019*	-0.27 (-0.63-0.09)	0.147
Adjusted R²	0.0533		0.04871	
F-statistic	20.52 on 6 and 2074 DF		18.49 on 6 and 2043 DF	

Reference group is Class 1 *stable high*. Models control for baseline sociodemographic characteristics. Subjective well-being measured on a 0–100 scale, BMI as continuous outcome. Estimates are unstandardised. Statistical significance is indicated by * p < 0.05, ** p < 0.01, and *** p < 0.001.

Differences in BMI (see Table 7) between classes are small, though individuals experiencing the *late drop* trajectory have an on average one point higher BMI, or – assuming an average size of 170 centimetres – weigh about 2.8 kg more. Females are markedly lighter in relation to size, as are those that attended academic track schools, though to a lesser extent. Furthermore, the intercept of 24.61 for BMI indicates that most young adults are close to the borderline of being overweight.

Regarding self-rated health (see Table 8), individuals experiencing either the *early drop* or *late drop* in subjective well-being in adolescence or *catching up* are at least twice as likely to report poor health than those with *stable high* trajectories. Females and individuals that attended academic track schools also more often report better health. Family type and parental income seem to have unexpected unfavourable associations, although to a minor extent that fails to reach statistical significance.

**Table 8:** Logistic regression analysis: well-being paths as predictors of health and health behaviour

	Poor self-rated health		Smoking status		Alcohol consumption	
	OR (95%-CI)	*p*-value	OR (95%-CI)	*p*-value	OR (95%-CI)	*p*-value
Constant	0.15 (0.11–0.21)	0.000***	0.56 (0.43–0.73)	0.000***	0.4 (0.31–0.53)	0.000***
Class ^Ref=class 1^						
Class 2 *early drop*	2.35 (1.66–3.3)	0.000***	1.83 (1.35–2.47)	0.000***	0.81 (0.58–1.13)	0.228
Class 3 *late drop*	2.87 (1.9–4.28)	0.000***	1.61 (1.09–2.35)	0.016*	0.69 (0.44–1.06)	0.103
Class 4 *catching up*	2.15 (1.02–4.15)	0.031*	1.45 (0.76–2.65)	0.237	0.53 (0.24–1.07)	0.097
Gender ^Ref=male^	1.67 (1.3–2.15)	0.000***	0.51 (0.41–0.62)	0.000***	0.4 (0.33–0.5)	0.000***
School ^Ref=low^	0.72 (0.56–0.94)	0.014*	0.8 (0.65–0.99)	0.039*	1.09 (0.88–1.35)	0.422
Family ^Ref=non−nuclear family^	0.86 (0.66–1.15)	0.303	0.71 (0.57–0.9)	0.004**	1.01 (0.8–1.28)	0.945
Parental income ^Ref=low^	0.83 (0.64–1.08)	0.166	1.04 (0.84–1.3)	0.709	1.48 (1.18–1.85)	0.001***

Reference group is Class 1 *stable high*. Models control for baseline sociodemographic characteristics. Statistical significance is indicated by * p < 0.05, ** p < 0.01, and *** p < 0.001.

Smoking (see Table 8) is also about one and a half to almost twice as common for those assigned to the *early drop*, *late drop* or *catching up* trajectory, though large confidence intervals point towards higher uncertainty, especially for those *catching up*. Furthermore, males are almost twice as likely to smoke, and smoking is also more prevalent among those from non-academic track schools or not growing up with both parents.

Problematic alcohol consumption (see Table 8) of at least one drink per week exhibits no significant associations with class membership, but results point towards a higher prevalence for those from the *stable high* trajectory. For gender and income, on the other hand, there are very clear associations, as males are more than twice as likely to drink often, and individuals with higher parental income are also more likely to exhibit patterns of problematic alcohol consumption.

## Discussion

### Summary

This study examined longitudinal data on subjective well-being from the National Educational Panel Study (NEPS) to identify trajectories of well-being throughout adolescence. Subjective well-being was assessed through validated self-report measures, including domains such as life satisfaction, health satisfaction, and emotional well-being. Latent class growth analysis (LCGA) and latent growth mixture modelling (LGMM) were employed to identify distinct trajectories of well-being from ages 11 to 21. These methods allowed for the grouping of individuals based on similar patterns of change over time while accounting for variability within and across classes. The final model selection was guided by statistical fit indices, theoretical considerations, and interpretability, culminating in the identification of four well-defined trajectories of well-being, each offering insights into the developmental pathways that adolescents experience as they transition into young adulthood.

The *stable high* trajectory is most common and characterised by consistently high levels of subjective well-being across adolescence, with minimal fluctuation. This trajectory serves as the reference point for understanding other patterns and represents the majority of adolescents. Adolescents in the *early drop* trajectory experienced a sharp decline in well-being during mid-adolescence, particularly around age 15. This decline coincided with key developmental challenges such as the onset of puberty, increasing academic demands and social transitions. However, well-being recovered toward the end of adolescence. The *late drop* trajectory began with stable well-being in early adolescence but experienced a significant decline during late adolescence, with a low point around age 17. Unlike the *early drop* group, these adolescents did not fully recover by the end of the observed period. The fewest adolescents were classified into the *catching up* trajectory and reported very low well-being at the start of adolescence but demonstrated significant improvement over time, reaching levels comparable to the *stable high* group by young adulthood.

Socio-demographic and socio-economic factors played a significant role in determining trajectory membership. Adolescents from higher-income households and nuclear families were more likely to follow the *stable high* trajectory, while those from single-parent or step-family households and lower-income families were more likely to fall into the *catching up* or *early drop* groups. Gender differences were also observed, with males more commonly represented in the *catching up* trajectory and females more likely to experience early or late drops.

The trajectories significantly predicted health-related outcomes in young adulthood. For instance, individuals in the *stable high* group reported better self-rated health, lower smoking rates, and healthier BMIs.

Conversely, those in the *late drop* group exhibited higher risks of adverse health behaviours and poorer health. The *early drop* and *catching up* groups demonstrated intermediate outcomes, with partial recovery in well-being but lingering vulnerabilities in health behaviours.

These results highlight the diverse pathways of adolescent well-being and their implications for long-term health-related outcomes, emphasising the importance of early interventions and supportive environments to promote positive trajectories and mitigate risks associated with negative ones.

### Integration into existing literature

The findings regarding the overall trajectories of well-being throughout adolescence are found to mostly align with prior research. The predominance of the *stable high* trajectory is in line with previous studies reporting that most adolescents maintain relatively stable and favourable well-being during this life stage, as documented in the UK [28], Finland [33] and international reviews [5, 6]. Furthermore, significant changes in well-being have been found to manifest primarily later in adulthood, as observed in international panel data [73].

The *early drop* and *late drop* trajectories, which represent significant proportions of the sample, relate to analogous findings in prior research from Germany [25, 37] and Finland [30, 33], where an overall decrease in well-being and life satisfaction during adolescence has been reported. While the subsequent increase in well-being towards the end of adolescence and when leaving school has been observed in some national contexts [32, 33], as well as in an international scoping review [74] this pattern does not emerge consistently across all datasets [28].

However, the relatively small proportion of adolescents *catching up* and starting off with a markedly lower well-being and experiencing a profound increase, up to a level that can no longer be distinguished from the other groups by the end of adolescence, is rarely reported by this previous research. Some studies (e.g., [28]) have suggested a lack of significant upward trends in well-being during late adolescence. Methodological differences, such as sample composition, measurement tools, and cultural contexts, may account for these inconsistencies. These findings resonate with resilience research from Germany [75] and international contexts [76], which emphasise the role of protective factors in facilitating recovery from adversity. While prior studies have identified resilience trajectories, the *catching up* group in this study offers a novel perspective by linking specific socio-demographic predictors, notably being male, attending non-academic school tracks and living in a single-parent or step-parent family, to a pattern of profound recovery from initially low well-being. This suggests that even adolescents starting from a disadvantaged position can achieve parity with their peers, although the small number of individuals in this group warrants a cautious interpretation of these findings, as also discussed in the Limitations section.

The role of socio-demographic and socio-economic predictors in determining trajectory membership aligns with prior research from Germany [13] and international contexts [23], emphasizing the associations between structural inequalities on adolescent well-being. Studies have consistently shown that factors such as parental education, household income, and family composition influence emotional and psychological development during adolescence. From a psychosocial stress perspective, these disadvantages can lead to chronically elevated stress levels, particularly in single-parent or step-family households. This stress, especially when experienced during sensitive developmental periods, may disrupt emotional regulation and has been found to relate to declining well-being over time in research from the United States [77, 78]. The present study adds to these findings by indicating that adolescents from nuclear families and higher-income households are more likely to follow the *stable high* trajectory, whereas those from single-parent or step-family households are at greater risk of experiencing well-being declines as also found in the US [79]. These results underscore the potential of family-centred interventions that strengthen financial and social resources to reduce the risk of adverse developmental pathways.

Gender differences also played a significant role in trajectory membership, partially aligning with and at times contradicting previous findings. While prior research from Spain suggests that girls typically report lower well-being and are at greater risk for early declines [27], our results indicate that females in this German cohort were more frequently found in the *late drop* trajectory rather than the *early drop* group. While differences in pubertal timing have been linked to increased psychological distress among girls [80], a pattern more consistent with early adolescent declines in well-being, the later decline observed in the present study may instead reflect additional or cumulative processes unfolding during late adolescence, such as increasing academic pressures [81], gender-specific societal expectations [82, 83], or prolonged exposure to stressors [14].

Consistent with this perspective, research from the UK [39] and Germany [37] points to a more pronounced and enduring disadvantage for adolescent girls in terms of mental health outcomes, with long-term implications for well-being and social integration.

These findings suggest that gender-specific interventions may be necessary to address the unique challenges faced by female adolescents, particularly during late adolescence when well-being appears to decline more substantially.

Similarly, health-related outcomes associated with well-being trajectories provide critical insights into the long-term implications of adolescent well-being. Prior research has found that subjective well-being in adolescence is strongly linked to later physical health, health-risk behaviours, and overall life satisfaction [84]. Our results resonate with these findings, indicating that individuals in the late-drop trajectory exhibit higher risks of adverse health behaviours, including higher likelihood of smoking and elevated BMI [23]. Conversely, those in the *stable high* and *catching up* groups tend to maintain better health-related outcomes, reinforcing the notion that adolescent well-being plays a foundational role in shaping long-term health trajectories [20]. These findings highlight the potential of early interventions that not only address mental health but also promote healthy lifestyle habits which may persist into adulthood.

### Strengths and limitations

This study benefits from a large, nationally representative dataset with yearly follow-ups, ensuring high external validity. The application of advanced latent growth mixture modelling (LGMM) allowed for a nuanced analysis of well-being trajectories, making it one of the first studies to compare predicted and self-reported values in this manner. The robustness of the results across different model specifications further strengthens their reliability. Additionally, the subjective well-being measures used are comparable with instruments in other large-scale panel studies, facilitating cross-study validation.

Several limitations must be acknowledged. First, high attrition, common in longitudinal research, may introduce biases despite efforts to address missing data under the Missing at Random (MAR) assumption. As in all longitudinal observational studies, the MAR assumption cannot be empirically verified, and missingness depending on unobserved well-being trajectories cannot be ruled out. Second, many time-variant variables were treated as time-invariant due to modelling constraints, potentially overlooking dynamic influences. The links between changes in, for example, family type or school environment and children’s health and well-being should be, and are being, specifically investigated. Third, the *catching up* class comprises only 2.8% of the sample, resulting in a pronounced imbalance relative to the *stable high* group (78.3%). Consequently, comparisons involving this class should be interpreted with caution, as the small class size may compromise the stability of parameter estimates and susceptibility to bias. That being said, it is noteworthy that this pattern was also identified in all other models when examining at least four classes and was always characterised by initially very low levels of subjective well-being at the beginning of adolescence, catching up toward the end, corroborating our decision to retain it as a distinct trajectory class. Fourth, measurement invariance was not fully established, though strict invariance is not necessarily required for within-individual trajectory analyses. Nevertheless, deviations from full invariance should be considered, particularly when making cross-cohort comparisons.

Moreover, the study’s focus on Germany limits generalizability to other socio-cultural contexts. While the dataset is nationally representative, future research should incorporate international comparisons to explore whether similar well-being trajectories emerge in different contexts. Finally, reliance on self-reported well-being measures introduces potential biases, including social desirability and recall bias. Although validated instruments were used, self-perceptions may not fully align with objective indicators, requiring cautious interpretation of the results.

### Future research directions

Building upon the current findings, future research should explore additional predictors and moderators of well-being trajectories to identify factors that amplify resilience or exacerbate vulnerability. For example, examining personality traits, coping strategies, or peer influences could shed light on individual-level differences in trajectory membership. Furthermore, a focus on environmental factors, such as school climate or neighbourhood context, could enhance understanding of the role external systems play in shaping adolescent experiences. In particular, more research is needed to investigate whether and which predictors determine the low starting values of the *catching up* group, in order to implement early interventions that can effectively respond to possible adverse childhood experiences. Developing a tool to retrospectively survey these long-term experiences during adolescence could help identify sensitive periods or conditions for targeted support.

Expanding the scope of research to include cross-cultural comparisons would provide insights into the universality of these trajectories across diverse socio-cultural contexts. Such efforts could help disentangle cultural norms and systemic influences on adolescent well-being. Additionally, longitudinal studies extending beyond young adulthood are needed to determine the long-term associations of adolescent well-being trajectories on career success, relationships, and mental health in later life.

Methodologically, future work should consider integrating mixed methods to provide a more holistic understanding of adolescent well-being. Combining quantitative approaches with qualitative insights from interviews or focus groups could unravel nuanced experiences that enrich trajectory-based models.

Finally, research exploring intervention efficacy, particularly for high-risk groups like those in the early- and late-drop trajectories, would be instrumental in translating these findings into actionable programs and policies.

## Conclusions

This study sheds light on the developmental pathways of subjective well-being during adolescence, identifying four distinct trajectories that illuminate periods of both resilience and vulnerability. By integrating socio-demographic and socio-economic predictors, the findings highlight the intricate interplay between individual and contextual factors in shaping adolescent experiences. These trajectories not only predict important health-related outcomes in young adulthood but also elucidate the need for targeted interventions during key developmental periods.

In particular, the early- and late-drop trajectories reflect periods of heightened vulnerability that are well-documented in the literature. Mid-adolescence, associated with the early-drop group, often coincides with increased academic pressures and social reorientation, aligning with previous findings that document significant declines in well-being during this stage [31, 85, 86]. Similarly, the late-drop trajectory appears to be shaped by stressors related to the transition out of secondary education – such as uncertainty about future roles and increased autonomy demands – which have been identified as significant junctures for adolescent mental health [87]. These declines underscore the need for targeted support during these phases to buffer the adverse effects of stress and prevent long-term consequences for psychological well-being.

The recovery observed in the *catching up* group, although to be interpreted with caution, is less frequently reported in existing research and points toward the presence of resilience processes, likely supported by protective factors such as social support, stable routines, or identity consolidation. Investigating the early disadvantages and later adaptive mechanisms of this group may provide important insights for intervention design, particularly for adolescents who enter adolescence with markedly low levels of well-being but exhibit signs of subsequent recovery. Such insights could inform the timing and focus of interventions, both preventive and therapeutic, during early and mid-adolescence, for example by strengthening protective factors such as social support, coping skills, or access to mental health resources before well-being declines consolidate into more severe psychopathological conditions in adulthood [39, 88].

The findings emphasize the importance of equitable access to resources and supportive environments, especially for adolescents exposed to early disadvantage. In light of the observed gender differences in trajectory membership, interventions may also benefit from being gender-sensitive, for instance by addressing gender-specific stressors and societal expectations that become increasingly salient during adolescence. Targeted and timely interventions—implemented at the level of schools, families, or community services— may not only reduce suffering in adolescence but also contribute to improved health and well-being across the life course.

Finally, the study’s methodological rigor and longitudinal scope contribute to a growing understanding of adolescent well-being. By addressing current limitations and building upon these findings, future research can help shape more inclusive and effective public health strategies that promote healthier trajectories for all adolescents. Adolescence is a window of both risk and opportunity and understanding well-being trajectories can guide interventions that prevent lasting harm and foster healthier life paths.

## Supplementary Information

Below is the link to the electronic supplementary material.

Additional file 1: Latent Class Growth Analysis. Characteristics and criteria for increasing number of classes (K=1-7) using linear structure model including transition dummy and fixed intercept. Additional file 2: Latent Class Growth Analysis. Observed and predicted values for increasing number of classes (K=1-7) using linear structure model including transition dummy and fixed intercept. Additional file 3: Latent Class Growth Analysis. Characteristics and criteria for increasing number of classes (K=1-7) using quadratic structure model including transition dummy and fixed intercept. Additional file 4: Latent Class Growth Analysis. Observed and predicted values for increasing number of classes (K=1-7) using quadratic structure model including transition dummy and fixed intercept. Additional file 5: Latent Class Growth Analysis. Characteristics and criteria for increasing number of classes (K=1-7) using cubic structure model including transition dummy and fixed intercept. Additional file 6: Latent Class Growth Analysis. Observed and predicted values for increasing number of classes (K=1-7) using cubic structure model including transition dummy and fixed intercept. Additional file 7: Latent Growth Mixture Model. Characteristics and criteria for increasing number of classes (K=1-7) using linear structure model including transition dummy and random intercept. Additional file 8: Latent Growth Mixture Model. Observed and predicted values for increasing number of classes (K=1-7) using quadratic structure model including transition dummy and random intercept. Additional file 9: Latent Growth Mixture Model. Characteristics and criteria for increasing number of classes (K=1-7) using quadratic structure model including transition dummy and random intercept. Additional file 10: Latent Growth Mixture Model. Observed and predicted values for increasing number of classes (K=1-7) using quadratic structure model including transition dummy and random intercept. Additional file 11: Latent Growth Mixture Model. Characteristics and criteria for increasing number of classes (K=1-7) using cubic structure model including transition dummy and random intercept. Additional file 12: Latent Growth Mixture Model. Observed and predicted values for increasing number of classes (K=1-7) using cubic structure model including transition dummy and random intercept.

## Data Availability

The data that support the findings of this study are not publicly available due to data protection regulations and contractual restrictions with the data provider. The authors do not have permission to share the data. Access to these data requires application and approval by the data provider.This paper uses data from the National Educational Panel Study (NEPS): Starting Cohort Grade 5, doi:10.5157/NEPS: SC3:10.0.0. From 2008 to 2013, NEPS data was collected as part of the Framework Program for the Promotion of Empirical Educational Research funded by the German Federal Ministry of Education and Research (BMBF). As of 2014, NEPS is carried out by the Leibniz Institute for Educational Trajectories (LIfBi) at the University of Bamberg in cooperation with a nationwide network.

## Abbreviations

SES: Socioeconomic status
NEPS: National Educational Panel Study
SC3: NEPS Starting Cohort 3
BMI: Body mass index
LCGA: Latent class growth analysis
LGMM: Latent growth mixture modelling
BIC: Bayesian Information Criterion
SABIC: Sample-Adjusted Bayesian Information Criterion
GRoLTS: Guidelines for Reporting on Latent Trajectory Studies
MAR: Missing at Random
