# Supplementary material for: Longitudinal patterns of adolescent well-being and associations with health-related outcomes in young adulthood: a cohort study using latent growth mixture modelling
Source: Child Adolesc Psychiatry Ment Health. 2026 May 23;20:83. doi: 10.1186/s13034-026-01100-w (PMC13267387; doi:10.1186/s13034-026-01100-w)

Additional file 1: Latent Class Growth Analysis. Characteristics and criteria for increasing number of classes (K=1-7) using linear structure model including transition dummy and fixed intercept.

| G | loglik | npm | BIC | SABIC | entropy | Class membership (%) | | | | | | |
|---|---|---|---|---|---|---|---|---|---|---|---|---|
| | | | | | | 1 | 2 | 3 | 4 | 5 | 6 | 7 |
| 1 | -223710.6907 | 4 | 447457.4182 | 447444.7069 | 1 | 100 | | | | | | |
| 2 | -218007.3703 | 8 | 436086.8142 | 436061.3917 | 0.709 | 83 | 17 | | | | | |
| 3 | -216758.2272 | 12 | 433624.5648 | 433586.4311 | 0.779 | 79.5 | 3.6 | 16.9 | | | | |
| 4 | -215976.3398 | 16 | 432096.8268 | 432045.9818 | 0.794 | 78.3 | 2.8 | 7.8 | 11.1 | | | |
| 5 | -215548.5508 | 20 | 431277.2857 | 431213.7295 | 0.721 | 3.5 | 18.2 | 66.6 | 9 | 2.7 | | |
| 6 | -215230.5533 | 24 | 430677.3274 | 430601.06 | 0.708 | 4.3 | 3.8 | 6.4 | 60.3 | 23.3 | 1.9 | |
| 7 | -215064.6772 | 28 | 430381.612 | 430292.6334 | 0.723 | 60.9 | 1.8 | 3.7 | 7.7 | 21.6 | 1.2 | 3 |

Notes: G = number of groups. loglik = log-likelihood. npm = number of parameters. BIC = Bayesian Information Criterion. SABIC = Sample-Adjusted Bayesian Information Criterion. % G = percentage of identified groups in given model.

Additional file 2: Latent Class Growth Analysis. Observed and predicted values for increasing number of classes (K=1-7) using linear structure model including transition dummy and fixed intercept.

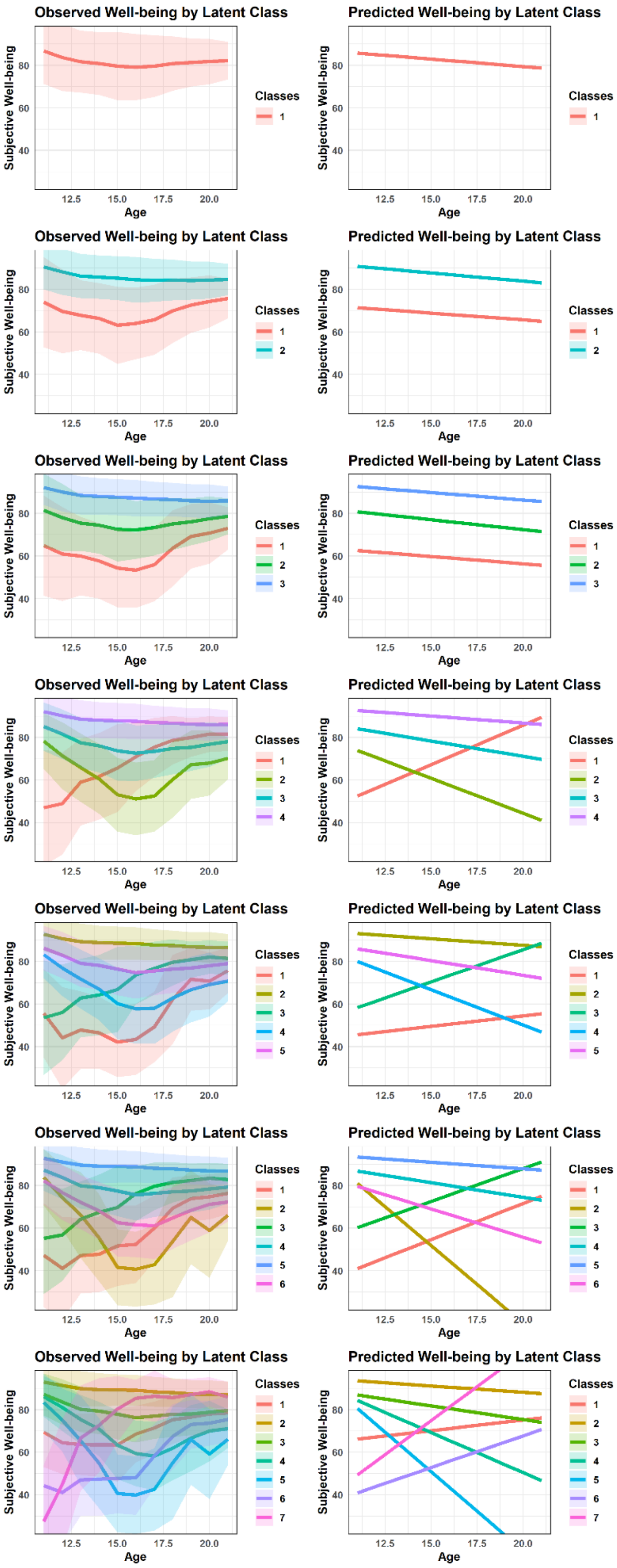

Additional file 3: Latent Class Growth Analysis. Characteristics and criteria for increasing number of classes (K=1-7) using quadratic structure model including transition dummy and fixed intercept.

| **G** | **loglik** | **npm** | **BIC** | **SABIC** | **entropy** | **Class membership (%)** | | | | | | |
|---|---|---|---|---|---|---|---|---|---|---|---|---|
| | | | | | | **1** | **2** | **3** | **4** | **5** | **6** | **7** |
| 1 | -223635,209 | 5 | 447315,464 | 447299,575 | 1 | 100 | | | | | | |
| 2 | -217851,186 | 10 | 435792,464 | 435760,686 | 0,709 | 83 | 17 | | | | | |
| 3 | -216585,828 | 15 | 433306,795 | 433259,128 | 0,779 | 79,5 | 3,6 | 16,9 | | | | |
| 4 | -215318,625 | 20 | 430817,434 | 430753,877 | 0,794 | 78,3 | 2,8 | 7,8 | 11,1 | | | |
| 5 | -214914,871 | 25 | 430054,971 | 429975,526 | 0,721 | 3,5 | 18,2 | 66,6 | 9 | 2,7 | | |
| 6 | -214559,981 | 30 | 429390,238 | 429294,904 | 0,708 | 4,3 | 3,8 | 6,4 | 60,3 | 23,3 | 1,9 | |
| 7 | -214334,698 | 35 | 428984,717 | 428873,494 | 0,723 | 60,9 | 1,8 | 3,7 | 7,7 | 21,6 | 1,2 | 3 |

Notes: G = number of groups. loglik = log-likelihood. npm = number of parameters. BIC = Bayesian Information Criterion. SABIC = Sample-Adjusted Bayesian Information Criterion. % G = percentage of identified groups in given model.

Additional file 4: Latent Class Growth Analysis. Observed and predicted values for increasing number of classes (K=1-7) using quadratic structure model including transition dummy and fixed intercept.

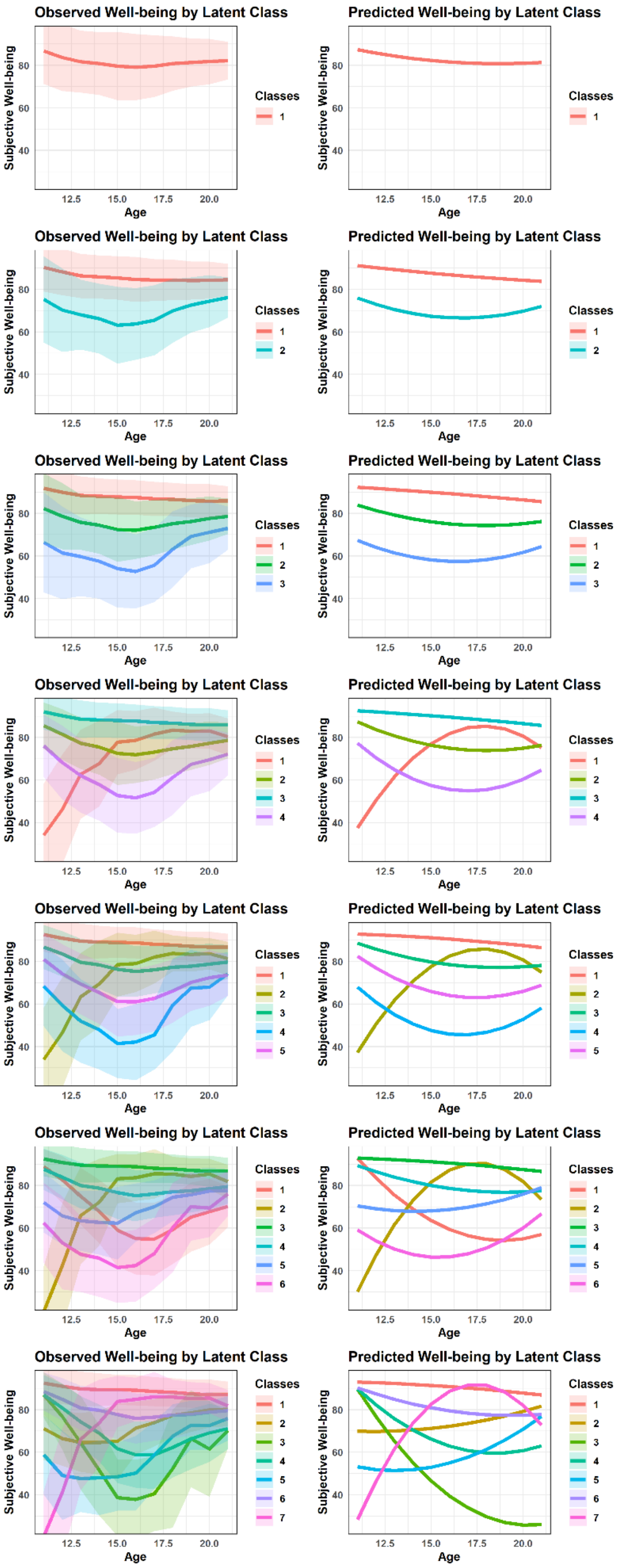

Additional file 5: Latent Class Growth Analysis. Characteristics and criteria for increasing number of classes (K=1-7) using cubic structure model including transition dummy and fixed intercept.

| G | loglik | npm | BIC | SABIC | entropy | Class membership (%) | | | | | | |
|---|---|---|---|---|---|---|---|---|---|---|---|---|
| | | | | | | 1 | 2 | 3 | 4 | 5 | 6 | 7 |
| 1 | -223635,209 | 5 | 447315,464 | 447299,575 | 1 | 100 | | | | | | |
| 2 | -217851,186 | 10 | 435792,464 | 435760,686 | 0,709 | 83 | 17 | | | | | |
| 3 | -216585,828 | 15 | 433306,795 | 433259,128 | 0,779 | 79,5 | 3,6 | 16,9 | | | | |
| 4 | -215318,625 | 20 | 430817,434 | 430753,877 | 0,794 | 78,3 | 2,8 | 7,8 | 11,1 | | | |
| 5 | -214914,871 | 25 | 430054,971 | 429975,526 | 0,721 | 3,5 | 18,2 | 66,6 | 9 | 2,7 | | |
| 6 | -214559,981 | 30 | 429390,238 | 429294,904 | 0,708 | 4,3 | 3,8 | 6,4 | 60,3 | 23,3 | 1,9 | |
| 7 | -214334,698 | 35 | 428984,717 | 428873,494 | 0,723 | 60,9 | 1,8 | 3,7 | 7,7 | 21,6 | 1,2 | 3 |

Notes: G = number of groups. loglik = log-likelihood. npm = number of parameters. BIC = Bayesian Information Criterion. SABIC = Sample-Adjusted Bayesian Information Criterion. % G = percentage of identified groups in given model.

Additional file 6: Latent Class Growth Analysis. Observed and predicted values for increasing number of classes (K=1-7) using cubic structure model including transition dummy and fixed intercept.

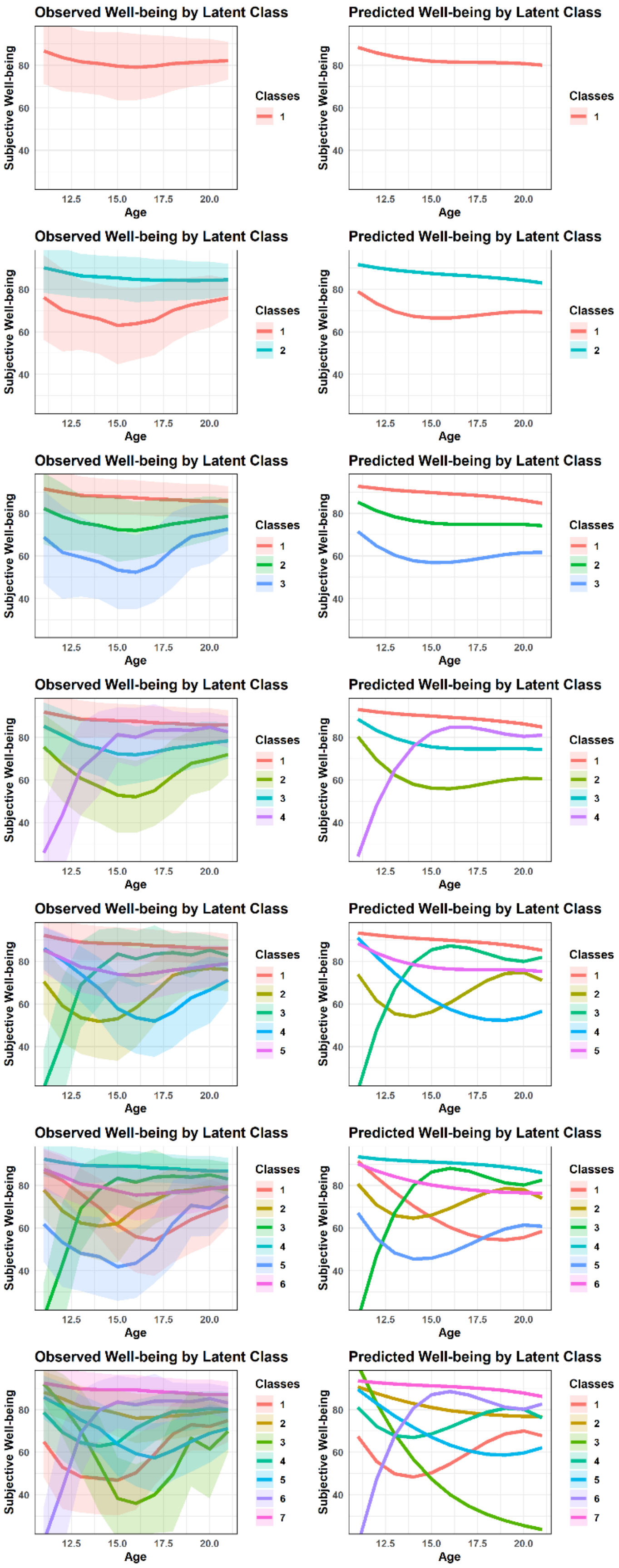

Additional file 7: Latent Growth Mixture Model. Characteristics and criteria for increasing number of classes (K=1-7) using linear structure model including transition dummy and random intercept.

| **G** | **loglik** | **npm** | **BIC** | **SABIC** | **entropy** | **Class membership (%)** | | | | | | |
|---|---|---|---|---|---|---|---|---|---|---|---|---|
| | | | | | | **1** | **2** | **3** | **4** | **5** | **6** | **7** |
| 1 | -217521,938 | 5 | 435088,922 | 435073,033 | 1 | 100 | | | | | | |
| 2 | -216300,259 | 10 | 432690,609 | 432658,831 | 0,709 | 83 | 17 | | | | | |
| 3 | -215257,475 | 15 | 430650,088 | 430602,421 | 0,779 | 79,5 | 3,6 | 16,9 | | | | |
| 4 | -214994,259 | 20 | 430168,701 | 430105,145 | 0,794 | 78,3 | 2,8 | 7,8 | 11,1 | | | |
| 5 | -214802,981 | 25 | 429831,193 | 429751,747 | 0,721 | 3,5 | 18,2 | 66,6 | 9 | 2,7 | | |
| 6 | -214681,908 | 30 | 429634,092 | 429538,758 | 0,708 | 4,3 | 3,8 | 6,4 | 60,3 | 23,3 | 1,9 | |
| 7 | -214787,981 | 35 | 429891,284 | 429780,061 | 0,723 | 60,9 | 1,8 | 3,7 | 7,7 | 21,6 | 1,2 | 3 |

Notes: G = number of groups. loglik = log-likelihood. npm = number of parameters. BIC = Bayesian Information Criterion. SABIC = Sample-Adjusted Bayesian Information Criterion. % G = percentage of identified groups in given model.

Additional file 8: Latent Growth Mixture Model. Observed and predicted values for increasing number of classes (K=1-7) using quadratic structure model including transition dummy and random intercept.

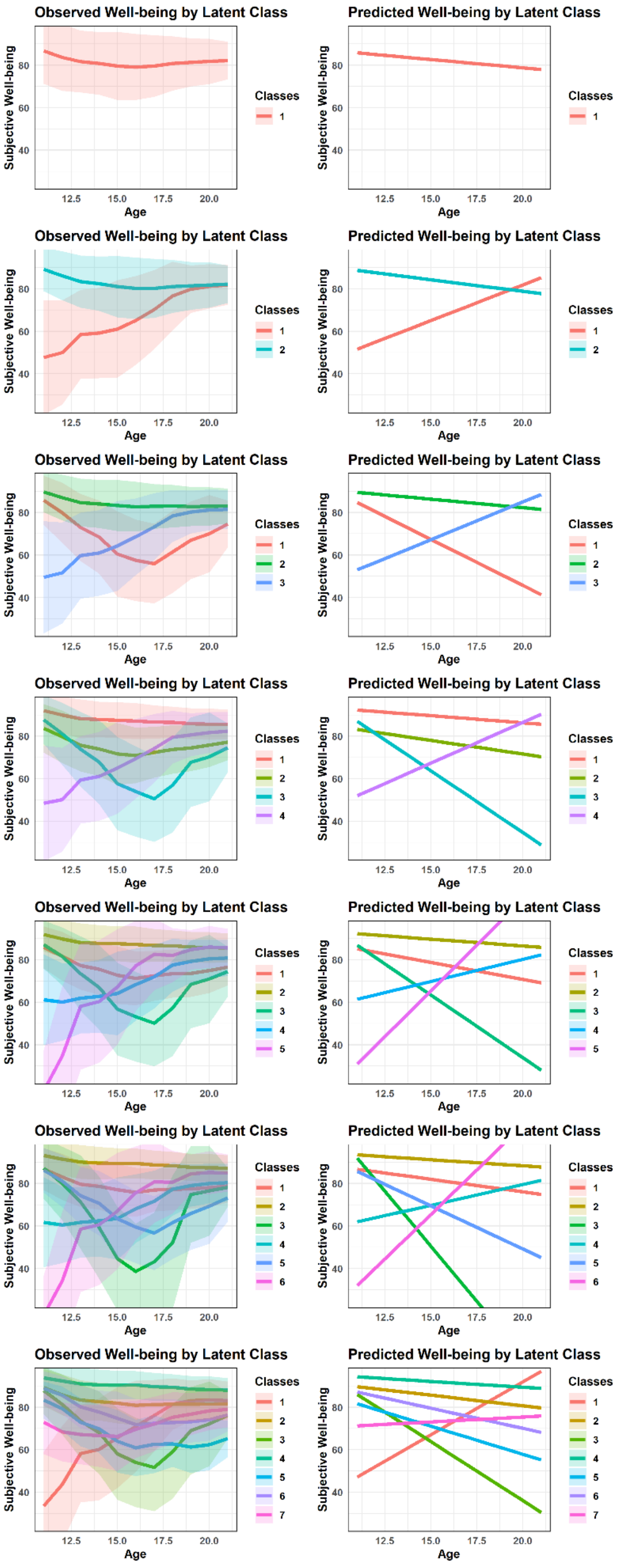

Additional file 9: Latent Growth Mixture Model. Characteristics and criteria for increasing number of classes (K=1-7) using quadratic structure model including transition dummy and random intercept.

| G | loglik | npm | BIC | SABIC | entropy | Class membership (%) | | | | | | |
|---|---|---|---|---|---|---|---|---|---|---|---|---|
| | | | | | | 1 | 2 | 3 | 4 | 5 | 6 | 7 |
| 1 | -217455,451 | 6 | 434964,958 | 434945,891 | 1 | 100 | | | | | | |
| 2 | -216322,012 | 12 | 432752,134 | 432714 | 0,709 | 83 | 17 | | | | | |
| 3 | -214761,749 | 18 | 429685,663 | 429628,463 | 0,779 | 79,5 | 3,6 | 16,9 | | | | |
| 4 | -214369,05 | 24 | 428954,321 | 428878,054 | 0,794 | 78,3 | 2,8 | 7,8 | 11,1 | | | |
| 5 | -214082,029 | 30 | 428434,334 | 428338,999 | 0,721 | 3,5 | 18,2 | 66,6 | 9 | 2,7 | | |
| 6 | -213908,91 | 36 | 428142,151 | 428027,75 | 0,708 | 4,3 | 3,8 | 6,4 | 60,3 | 23,3 | 1,9 | |
| 7 | -213799,878 | 42 | 427978,142 | 427844,674 | 0,723 | 60,9 | 1,8 | 3,7 | 7,7 | 21,6 | 1,2 | 3 |

Notes: G = number of groups. loglik = log-likelihood. npm = number of parameters. BIC = Bayesian Information Criterion. SABIC = Sample-Adjusted Bayesian Information Criterion. % G = percentage of identified groups in given model.

Additional file 10: Latent Growth Mixture Model. Observed and predicted values for increasing number of classes (K=1-7) using quadratic structure model including transition dummy and random intercept.

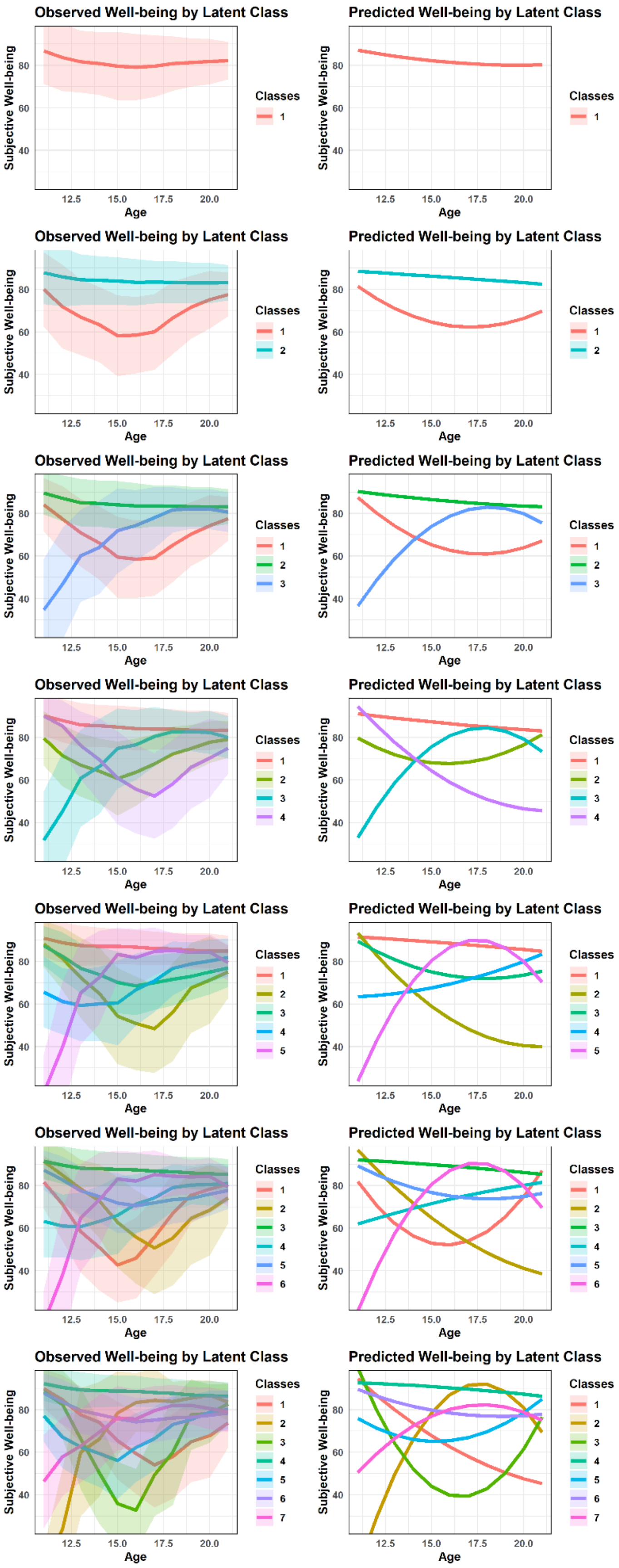

Additional file 11: Latent Growth Mixture Model. Characteristics and criteria for increasing number of classes (K=1-7) using cubic structure model including transition dummy and random intercept.

| G | loglik | npm | BIC | SABIC | entropy | Class membership (%) | | | | | | |
|---|---|---|---|---|---|---|---|---|---|---|---|---|
| | | | | | | 1 | 2 | 3 | 4 | 5 | 6 | 7 |
| 1 | -217455,451 | 6 | 434964,958 | 434945,891 | 1 | 100 | | | | | | |
| 2 | -216322,012 | 12 | 432752,134 | 432714 | 0,709 | 83 | 17 | | | | | |
| 3 | -214761,749 | 18 | 429685,663 | 429628,463 | 0,779 | 79,5 | 3,6 | 16,9 | | | | |
| 4 | -214369,05 | 24 | 428954,321 | 428878,054 | 0,794 | 78,3 | 2,8 | 7,8 | 11,1 | | | |
| 5 | -214082,029 | 30 | 428434,334 | 428338,999 | 0,721 | 3,5 | 18,2 | 66,6 | 9 | 2,7 | | |
| 6 | -213908,91 | 36 | 428142,151 | 428027,75 | 0,708 | 4,3 | 3,8 | 6,4 | 60,3 | 23,3 | 1,9 | |
| 7 | -213799,878 | 42 | 427978,142 | 427844,674 | 0,723 | 60,9 | 1,8 | 3,7 | 7,7 | 21,6 | 1,2 | 3 |

Notes: G = number of groups. loglik = log-likelihood. npm = number of parameters. BIC = Bayesian Information Criterion. SABIC = Sample-Adjusted Bayesian Information Criterion. % G = percentage of identified groups in given model.

Additional file 12: Latent Growth Mixture Model. Observed and predicted values for increasing number of classes (K=1-7) using cubic structure model including transition dummy and random intercept.

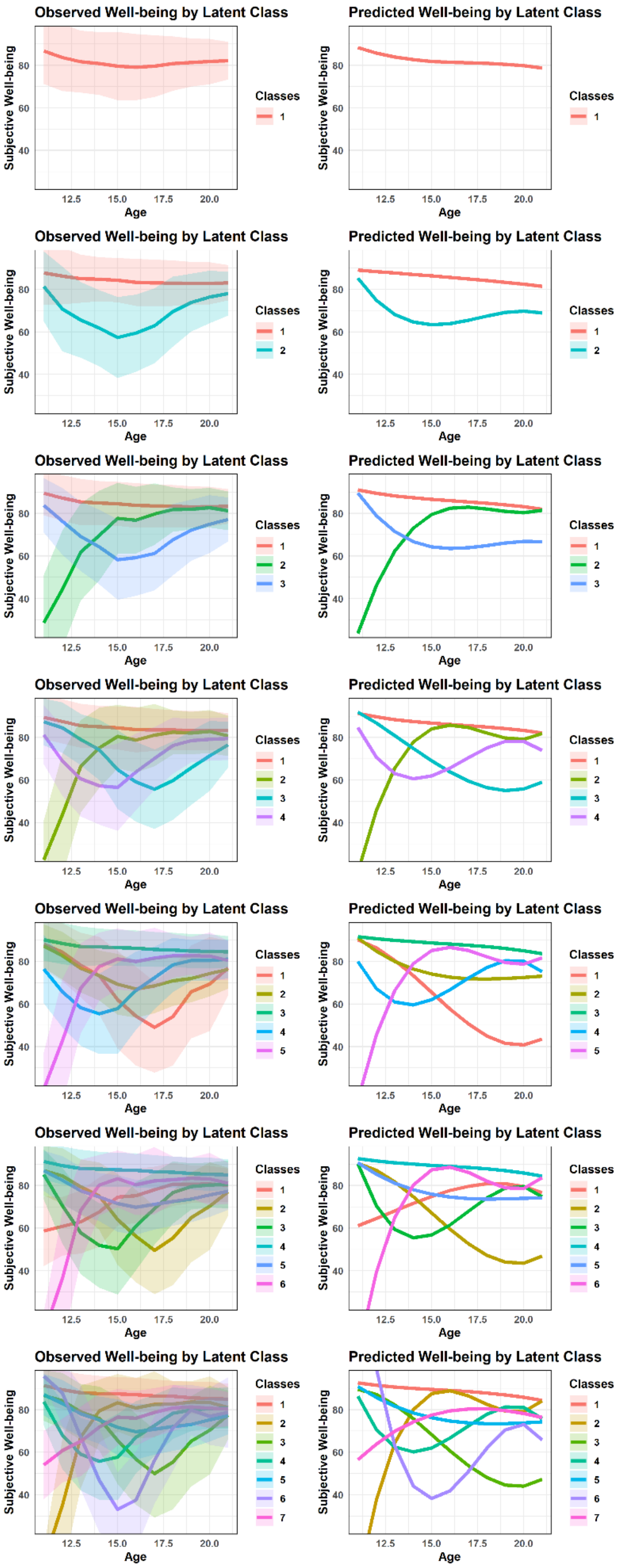

Supplement: Supplementary file 1 — Additional file 1: Latent Class Growth Analysis. Characteristics and criteria for increasing number of classes (K=1-7) using linear structure model including transition dummy and fixed intercept. Additional file 2: Latent Class Growth Analysis. Observed and predicted values for increasing number of classes (K=1-7) using linear structure model including transition dummy and fixed intercept. Additional file 3: Latent Class Growth Analysis. Characteristics and criteria for increasing number of classes (K=1-7) using quadratic structure model including transition dummy and fixed intercept. Additional file 4: Latent Class Growth Analysis. Observed and predicted values for increasing number of classes (K=1-7) using quadratic structure model including transition dummy and fixed intercept. Additional file 5: Latent Class Growth Analysis. Characteristics and criteria for increasing number of classes (K=1-7) using cubic structure model including transition dummy and fixed intercept. Additional file 6: Latent Class Growth Analysis. Observed and predicted values for increasing number of classes (K=1-7) using cubic structure model including transition dummy and fixed intercept. Additional file 7: Latent Growth Mixture Model. Characteristics and criteria for increasing number of classes (K=1-7) using linear structure model including transition dummy and random intercept. Additional file 8: Latent Growth Mixture Model. Observed and predicted values for increasing number of classes (K=1-7) using quadratic structure model including transition dummy and random intercept. Additional file 9: Latent Growth Mixture Model. Characteristics and criteria for increasing number of classes (K=1-7) using quadratic structure model including transition dummy and random intercept. Additional file 10: Latent Growth Mixture Model. Observed and predicted values for increasing number of classes (K=1-7) using quadratic structure model including transition dummy and random intercept. Additional file 11: L [file 13034_2026_1100_MOESM1_ESM.pdf]
